# Legitimacy from a Medical and Legal Standpoint

**Published:** 1910-06-25

**Authors:** 


### Medico-Legal Points.

### I.—LEGITIMACY FROM A MEDICAL AND LEGAL STANDPOINT.

In English law, a person born anywhere either in wedlock or within the *ultimum tempus pariendi* after its determination is legitimate; so also is anyone born out of wedlock whose parents afterwards marry, if both the law of the father's domicile at the birth and the law of his domicile at the marriage coincide in allowing *legitimatio per subsequens matrimonium* (2 Black. Com. 247. See *In re Goodman's* Trusts, 17 Ch. D. 266; *In re Grove Vaucher* v. *The Solicitor to the Treasury* (1888), 40 Ch. D. 216). Bastardy being by English law indelible the status of legitimacy cannot be obtained by any person born out of wedlock, if the father's domicile was English at the date of the birth. The rule is inflexible; legitimation, in its various forms, is unknown: adoption, recognition, the *legitimatio per subsequens matrimonium* itself, all but universal in Christendom as it is, have no place in our municipal law. Legitimacy is not dependent upon nationality or upon naturalisation or allegiance; it is a personal status accompanying its possessor wherever he goes. The comity of nations recognises those persons as legitimate everywhere who are legitimate by the law of their place of origin; and, for this purpose, the place of a person's origin is the domicile of the father. The domicile of the mother, the place of the child's birth, the *lex loci contractus* (provided the marriage be valid) are circumstances all alike immaterial in respect of legitimacy. The chief consequences of legitimacy have regard to property and to the conditions of its devolution, to heirship, to the operation of the Statutes of Distribution, and so forth. In these respects, with one remarkable exception, English law accords to all persons who are legitimate by the laws of their several domiciles every right which belongs to persons born in England of parents lawfully married. The exception is to be found in the rigid rule which denies to a person born before wedlock, but legitimated by subsequent marriage the full status of heirship. Heirship is determined by the *lex loci rei sitæ*; and accordingly, upon an intestacy, the real estate cannot be inherited except by a person who is not merely legitimate, but legitimate by reason of having been born in lawful wedlock. Full effect is given, in all other circumstances, to *legitimatio per subsequens matrimonium*; but where heirship is concerned, the principle is maintained that none but a person born in wedlock can inherit real estate.

### Legal Presumption of Legitimacy.

Every child born either in lawful matrimony, or within a period after the death of the husband in accordance with the natural duration of gestation, is considered by the English law to be the child of the husband, unless the contrary be made clearly to appear by medical or moral evidences, or by both combined. The common law of England was, according to Lord Coke, to this effect, that if a child was born during marriage, the husband being within the four seas of the realm, and no physical impossibility being proved, this child was legitimate. Access was presumed unless he could prove that he was beyond the four seas for above nine months previous to the birth. But the present state of the English law on the subject appears to be this. A child born during marriage is deemed illegitimate when, by good medical or other evidence, it is proved that it was impossible for the husband to be the father—whether from his being under the age of puberty, from his labouring under physical incapacity as a result of age or natural infirmity, or from the length of time which may have elapsed since he could have had intercourse, whether by reason of absence or death. The evidence to repel the presumption of legitimacy of a child born during wedlock must be strong, distinct, satisfactory and conclusive, and such as to produce a judicial conviction that the child was not procreated by the husband. Where, however, a husband and wife lived together for nine years without having a child, and then separated and never lived together again, and a child was born ten years after the separation, while the wife was in the habit of committing adultery with another man, which child was treated by the paramour as his own, and was called by his surname, and brought up by him, the child, notwithstanding possibility of access on the part of the husband, was held to be illegitimate. A mother's evidence is inadmissible to prove the legitimacy or illegitimacy of her child born during wedlock (*Atchley* v. *Sprigg*, 33 L.J. Ch. 345). The adultery of the wife, although throwing the greatest doubt upon the paternity of the child, is not conclusive. It must be shown the husband could not have had access which might result in paternity, otherwise the presumption of sexual intercourse between husband and wife, and consequently of legitimacy of the child, must prevail (*Gordon* v. *Gordon*, [1903] P. 141). There is a peculiar difference in relation to legitimacy, between the laws of England and Scotland. A child born of parents in Scotland before marriage is rendered legitimate by their subsequent marriage. In England the offspring is illegitimate, whether the parents marry or not after its birth; but under the Poor Law Act (4 & 5 Will. IV.) if a man marries a single woman having a child or children living, of whom possibly he is not the father, he is bound to maintain them as if they were his own, and born after marriage. At the same time the children are not legitimated by the marriage.

Every child born in wedlock is regarded, *primâ facie*, to be legitimate, unless impossibility of access or impossibility of intercourse can be proved. In other words, if the legitimacy of a child born in wedlock be questioned, it is for the opponents to prove the impossibility of legitimacy, and not for those who contend for its being legitimate to prove the same. In *Cope* v. *Cope* (5 C. & P. 604) the husband and wife lived fourteen miles apart, the wife having formed an illicit connection with another man. Both husband and wife regarded the last child to which she had given birth (the plaintiff) as illegitimate. On this, however, Baron Alderson said: "Lord Hardwicke decided that the mother could not be allowed to give evidence on such a point, as she could not discharge the husband of the birth of the child; and, *a fortiori*, the husband could not be permitted to discharge himself. Lord Mansfield and Lord Hardwicke both decided that illegitimacy could be proved only by the fact of there being no marriage, or by proof of non-access; and it was held, on the grounds of decency and morality, that the parties themselves should not be allowed to prove non-access after marriage." "If a child be born in marriage during the lifetime of the husband, that child, in law, is presumed to be legitimate. If a husband have access, and others at the same time have criminal intimacy with his wife, still a child born in such a case is legitimate in the eye of the law. But if the parties are living separately, and the wife is notoriously living in open adultery, although the husband have opportunities of access, yet under such circumstances it would be monstrous to suppose that he would avail himself of them. The legitimacy of a child so born could not, therefore, be established." The jury returned a verdict for the plaintiff that he was legitimate. In *Morris* v. *Davis* (8 L.J. Ch. 120) it was contended that the plaintiff was illegitimate, though born in wedlock. Husband and wife had voluntarily separated, but lived within a short distance of each other. The wife was living in adultery, and the child was born fourteen years after the separation. She even so far concealed the child's birth from her husband that he did not know of its existence for seventeen years. The Lord Chancellor, to whom the case was referred, said that although the husband and wife met occasionally, it so happened that none of these meetings would correspond with the time requisite for the birth of the child to make it legitimate, and, coupling with this the general bad conduct of the woman and her open adultery, he was led to regard the plaintiff as illegitimate. The case of *Plowes* v. *Bossey* (31 L.J. Ch. 681) was decided in favour of a child's legitimacy because, although the husband was in an asylum, his wife visited him at the asylum from time to time.

Non-access cannot be proved by the parties interested (namely, by the husband or wife), but must be determined by other evidence; in other words, married persons are not allowed to give evidence against the legitimacy of their children.

A child is legitimate if born during wedlock, although its conception may have occurred before wedlock. The justice of this is manifest, because the time and date of birth is definite, whilst the time of conception is indefinite.

A child born after the death of its mother (*e.g.*, by Cæsarean section) is legitimate, and at majority may claim the estate of which the mother was seised. The husband, however, in such case cannot claim, because his wife was dead, and the marriage therefore legally dissolved at the time of the birth.

A posthumous child is considered legitimate unless non-access, or impotence, or sterility on the part of the husband can be proved. A case of difficulty might occur as to paternity if a woman married immediately after the death of her first husband, and a child was born on or about the 280th day of the second marriage. Such a case would, however, be treated as a question of fact only—namely, of which husband was it the child? And in such case probably the evidence of the mother and of the second husband would be admissible to decide the question of paternity.

### Medical Questions Relating to Paternity.

Inquiries involving medical questions relating to disputed paternity may arise in the following cases:—

(1) In cases where a child is born after the husband's death (posthumous) or a little before his death, his health having failed some time previous to his decease. In such cases the following questions may arise (*a*) Was the supposed father in such a state of health as to be capable of begetting a child within the period named? (*b*) Do the size, the weight, and the development of the child agree with the possible period of gestation?

(2) In cases where a woman has a child either very shortly after marriage or after the prolonged absence of her husband.

In such cases two questions may arise: (*a*) Does the period of gestation, inferred from the size and development of the child, support the statements as to the possibility of marital access? (*b*) What is the earliest period at which a child is viable—in other words, capable of living—if due care be taken of it?

(3) In cases where a woman, having borne one child, gives birth after a short interval to a second. In such cases two questions may arise: (*a*) Is the case in question one of superfœtation? or (*b*) Were both children the issue of a single coitus?

(4) In cases where a woman marries immediately, either after the death of her husband or after a divorce, and a child is born soon after the second marriage. In such cases the following question may arise: Whether the child is the child of the deceased or divorced husband (as the case may be) or of the living or existing one.

(5) In cases where a woman, during the lifetime of her husband, pretends to have given birth to a child, and it is suspected that she is palming off, for the purpose of deception, the child of somebody else. In such cases the following questions will probably arise: (*a*) Has the woman ever given birth to a child? (*b*) Is her age and physical conformation consistent with her having given birth to the child in question? (*c*) Is it probable that a man so old or so feeble as the husband would be the father of a child? (*d*) Has the child any peculiar marks by which its parentage may be identified?

(6) In cases where a decree of nullity of marriage is sought on the ground that one of the parties is incapable of sexual intercourse.

### Term of Gestation.

By the common consent of mankind, the term of gestation is considered to be ten lunar months, or forty weeks, equal to nine calendar months and a week. This period has been adopted, because general observation, in cases which allow of accurate observation, has proved its correctness. It is not, however, denied that differences of one or two weeks have occurred. A question which requires consideration is, whether a child with all the characters of maturity can be born before the ordinary term of gestation. It has a direct bearing on the subject of legitimacy. A husband, for example, has been absent from his family, and at the end of seven or eight months after his return a full-grown healthy child is produced. Is the honour of the family to be impeached, or are we to allow this variation to be possible? There is an intrinsic difficulty connected with this question which should lead us to be tender in forming our opinions; and this originates from the variety observed in children when born at the full time. They differ in size, general appearance, healthiness, etc.; and sometimes, indeed, we know that eight months' children have been observed larger and healthier than those of nine months. The general appearance, then, should be noticed, but not too much relied on, in forming an unfavourable opinion.

There are many recorded cases of protracted delivery which have been made the subject of legal investigation. Godefroy, in his Notes on the Morals of Justinian, mentions a case where a widow was delivered fourteen months after the death of her husband, and her issue pronounced legitimate by the Parliament of Paris. It appeared that she had lived with the relatives of her husband during the whole period of widowhood; that they had never observed any impropriety in her conduct; and they also testified to the deep and constant grief she had manifested for the loss of her partner. The Parliament of Paris appears, indeed, to have adjudicated on numerous cases of protracted gestation. Foderé (vol. ii. p. 111) gives an abstract of twelve cases.

Thomas Bartholen (Foderé, vol. ii. p. 183) relates of a young girl at Leipsic, who, on accusing a person of having seduced her, was confined and strictly guarded. At the end of sixteen months she brought forth a child, which lived two days.

There are cases which deserve notice from the controversies to which they have given origin.

A female, the wife of a bookseller in Wolfenbuttel, was delivered thirteen months after the death of her husband. The individuals interested proposed to contest the legitimacy of the infant, but were deterred on account of her excellent character. So convinced was one Christopher Misnerus, who had acted as shopkeeper during her widowhood, of her virtue and probity, that he married her shortly after, and had two children by her, and each of them was born after a gestation of thirteen months. (Schlegel, vol. ii. p. 99 to 113, Wagner's Dissertation.)

Dalignac, chirurgeon major to the regiment of Asfeld, testified that, with three children which his wife had produced, the term in two had been thirteen and a half months, and in the third eleven months, and that he had recognised the existence of each of the pregnancies at four months and a half by the most infallible sign—the motion of the child (Foderé, vol. ii. pp. 185 to 189).

The following enlisted all the medical talent of France in its discussion. Charles ——, aged upwards of seventy-two years, married Renée, aged about thirty years, at the commencement of the year 1759. They were married nearly four years without having any issue. On October 17, 1762, he was taken ill with fever and violent oppression, which remained until his death. The last symptom was so severe that he was forced to sit in his bed, nor could he move without assistance. In addition to these, he was seized with a dry gangrene of the leg on the 21st; and with this accumulation of disease he gradually sank and died on November 17, aged seventy-six years. Renée had not slept in the chamber during his illness; but about three and a half months after his death she suggested that she was pregnant, and on October 3, 1763 (within four days of a year since the illness of her husband, and ten months and seventeen days after his death) she was delivered of a healthy, well-formed, and full-sized child. The opinion of Louis was asked on this case, and he declared that the offspring was illegitimate. Had he rested at this, even the advocates of protracted gestation might probably not have murmured, as the circumstances were rather too powerful for the interposition of their favourite doctrines. But he took occasion, in his consultation, to attack the opinion generally and to deny the possibility of the occurrence of such cases. Among the arguments which he adduces are the following: That the laws of nature on this subject are immutable; that the fœtus, at a fixed period, has received all the nourishment of which it is susceptible from the mother, and becomes, as it were, a foreign body; that married females are very liable to error in their calculations; that the decisions of tribunals in favour of protracted gestation cannot overturn a physical law; and, finally, that the virtue of females in these cases is a very uncertain guide for legal decisions. "If we admit," says he, "all the facts reported by ancient and modern authors of delivery from eleven to twenty-three months, it will be very commodious for females; and if so great a latitude is allowed for the production of posthumous heirs the collateral ones may in all cases abandon their hopes unless sterility be actually present." (Louis' "Mémoire contre Légitimité des Naissances prétendues tardives.) Mahon, in his chapter on this subject (Mahon, vol. i. pp. 183, 185, 196, 203) adds several remarks in confirmation of it. He observes that if the doctrine be true that the children of old people are longer in coming to maturity, it would have been confirmed by experience, which it is not. Grief also, and the depressing passions, are more apt to produce abortion than protracted gestation. He suggests that the menses may be suppressed not only from disease but from affections of the mind or accidental causes which do not immediately impair the health. If the doctrine be allowed, how shall we distinguish a delayed child from one that is born at nine months, and by what means are we to detect fraud in such cases?

